# Tetrodotoxin Oral Film Attenuates Depression in a Chronic Unpredictable Mild Stress Model in Mice

**DOI:** 10.3390/md24030094

**Published:** 2026-02-26

**Authors:** Jianlin He, Chao Tang, Siwen Niu, Qingqing Le, Lin Yu, Bihong Hong

**Affiliations:** 1 Third Institute of Oceanography, Ministry of Natural Resources, Xiamen 361005, China; 2 Technical Innovation Center for Exploitation of Marine Biological Resources, Ministry of Natural Resources, Xiamen 361005, China

**Keywords:** VGSCs, TTX, behavioral assessments, serotonin, acute toxicity

## Abstract

Depression remains a major global health challenge, with a significant proportion of patients failing to respond to conventional antidepressants. This study aimed to evaluate the potential antidepressant effects and toxicological profile of a novel tetrodotoxin (TTX) oral film formulation in a mouse model of chronic unpredictable mild stress (CUMS). Male C57BL/6J mice were subjected to CUMS and treated daily with TTX oral film at doses of 10, 20, and 40 μg/kg, with fluoxetine (18 mg/kg) serving as a positive control. Behavioral assessments, including sucrose preference test, open field test, forced swimming test, elevated plus maze, and novel object recognition, demonstrated that TTX oral film administration alleviated depression- and anxiety-like behaviors and improved cognitive function. Furthermore, TTX oral film treatment restored hippocampal serotonin levels, which were depleted in CUMS mice, and showed no adverse effects on organ indexes after long-term use. Toxicological evaluation through acute toxicity testing revealed an oral LD_50_ of 919 μg/kg, indicating a substantially improved safety profile compared to pure TTX and a wide therapeutic window. These findings suggest that the TTX oral film possesses significant antidepressant activity with favorable toxicological properties, supporting its potential as a novel and safe treatment for depression.

## 1. Introduction

Depression is a debilitating mental disorder that leads to substantial impairment in quality of life and functional capacity, and is recognized as a major cause of disability worldwide. Its growing prevalence underscores an urgent public health challenge [1]. The pathogenesis of depression involves a complex interplay of multiple biological, psychological, and environmental risk factors that converge to disrupt neurobiological homeostasis [2]. Current treatments for depression have expanded beyond traditional approaches to include rapid-acting biologics, targeted neuromodulation, and digital interventions, alongside established psychotherapy, pharmacotherapy, and electroconvulsive therapy [3]. In most cases, pharmacotherapy remains the most important and commonly used treatment for depression. Although an increasing number of antidepressant drugs are used in clinic, more than 30% of patients remain refractory to conventional antidepressants [4]. Therefore, the development of new types of antidepressant drugs has always been a hot topic in research.

Tetrodotoxin (TTX) is a potent neurotoxin that selectively inhibits voltage-gated sodium channels (VGSCs), thereby preventing action potential generation and neuronal conduction [5]. While its toxicity is well-documented, accumulating evidence supports therapeutic applications of TTX at non-toxic doses. In clinical trials, TTX has demonstrated efficacy in treating chronic pain conditions, including cancer-related pain [6] and chemotherapy-induced neuropathic pain [7]. Additionally, TTX can ameliorate heroin withdrawal syndrome [8]. Preclinical studies have further revealed analgesic, local anesthetic, and anti-arrhythmic properties [9], suggesting its broad potential for central nervous system modulation. However, there have been no reports of TTX, a VGSC-specific blocker, being used to treat depression.

From a toxicological perspective, formulating TTX into a safe and controllable dosage form is essential to mitigate its inherent risks. Oral films offer a promising delivery system that combines accurate dosing, rapid dissolution, mucoadhesion, and ease of administration without water. These features collectively enhance patient compliance, particularly in populations with swallowing difficulties or cognitive impairment [10]. This approach not only improves safety profiles through controlled release but also aligns with precision medicine goals in neuropharmacology.

Therefore, this study was designed not only to address a literature gap, but also to critically evaluate the therapeutic potential and safety of a novel TTX oral film formulation in a preclinical model of depression. By integrating toxicological rationale with drug delivery innovation, we aim to explore a new pathway for treating depression that may overcome limitations of current antidepressants, such as delayed onset, variable efficacy, and adverse side effects.

## 2. Results

### 2.1. TTX Oral Film Elevated the Appearance and Behavioral Score of the Chronic Unpredictable Mild Stress Model (CUMS) Mice

**Figure 1:**
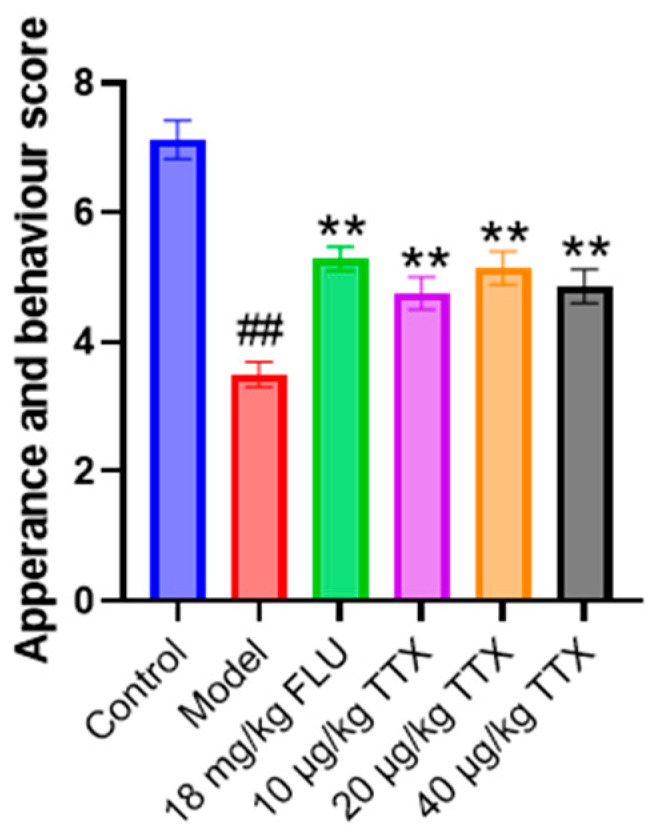
Appearance and behavioral scoring. ## *p* < 0.01 compared with the control group, ** *p* < 0.01 compared with the model group.

As shown in Figure 1, the appearance and behavioral scores of different experimental groups were evaluated. The model group scored significantly lower than the control group. For the groups with drug interventions, the fluoxetine (FLU) group showed a highly significant increase compared to the model group. The groups treated with different doses of TTX also presented significant elevated scores. These results suggest that TTX improved the appearance and behavioral performance in the CUMS model.

### 2.2. TTX Oral Film Alleviated Depression-like Behaviors in CUMS Mice

**Figure 2:**
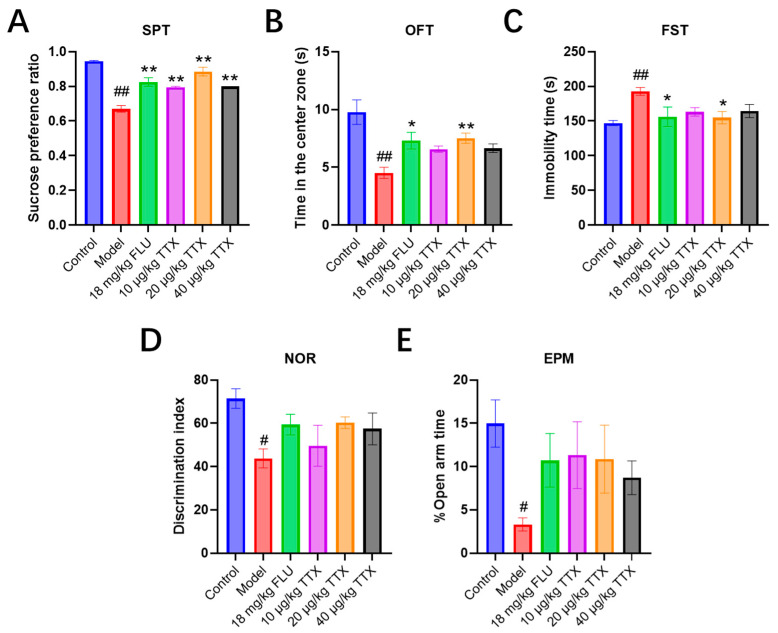
Behavioral assessments of TTX oral film (**A**) SPT, (**B**) OFT, (**C**) FST, (**D**) NOR, and (**E**) EPM. # *p* < 0.05, ## *p* < 0.01 compared with the control group; * *p* < 0.05, ** *p* < 0.01 compared with the model group.

In the sucrose preference test (SPT) (Figure 2A), the control group showed a high sucrose preference ratio (~0.9). The model group exhibited a significant reduction in sucrose preference (ratio ~ 0.7), indicative of anhedonia, a core symptom of depression. Treatment with 18 mg/kg FLU, 10 μg/kg TTX, 20 μg/kg TTX, and 40 μg/kg TTX all significantly increased sucrose preference compared to the model group, suggesting alleviation of anhedonia.

For the open field test (OFT, Figure 2B), the control group spent the longest time in the center zone (~10 s). The model group showed a marked decrease in center zone time (~4 s), reflecting increased anxiety-like behavior. FLU (18 mg/kg) and TTX (20 μg/kg) treatments significantly prolonged center zone time compared to the model group, indicating reduced anxiety-like behavior.

The results of the forced swimming test (FST) are shown in Figure 2C. The model group exhibited a significantly longer immobility time (~200 s) compared to the control group (~150 s), consistent with depressive-like behavior. Compared with the model group, FLU and all TTX dosed groups effectively shortened the immobility time, especially the 20 μg/kg TTX group, which shortened the immobility time by 19.69%, suggesting that TTX oral film has antidepressant-like effects.

The results of the novel object recognition (NOR) test are presented in Figure 2D. The control group exhibited a high discrimination index (~70), which reflects intact recognition memory. In contrast, the model group showed a notably lower discrimination index (~40), indicating cognitive impairment. Compared to the model group, treatments with FLU (18 mg/kg) and TTX (10–40 μg/kg) tended to increase the discrimination index, which suggests a potential improvement in recognition memory, though these changes did not reach statistical significance.

For the elevated plus maze (EPM, Figure 2E), the control group spent the most time in the open arms, with a percentage of approximately 14%. The model group showed a significant reduction in open arm time percentage (~3%), indicative of anxiety-like behavior. FLU (18 mg/kg) and TTX (10–40 μg/kg) treatments induced an upward trend in open arm time, which hints at a possible alleviation of anxiety-like behavior, though such improvements failed to achieve statistical significance.

In summary, TTX oral film treatments alleviated depressive-like (SPT, FST), anxiety-like (OFT, EPM), and cognitive impairment (NOR) behaviors in the model. The 20 μg/kg TTX group exhibited effects comparable to the positive control FLU (18 mg/kg).

### 2.3. TTX Oral Film Elevated Hippocampal Serotonin (5-HT) Content

**Figure 3:**
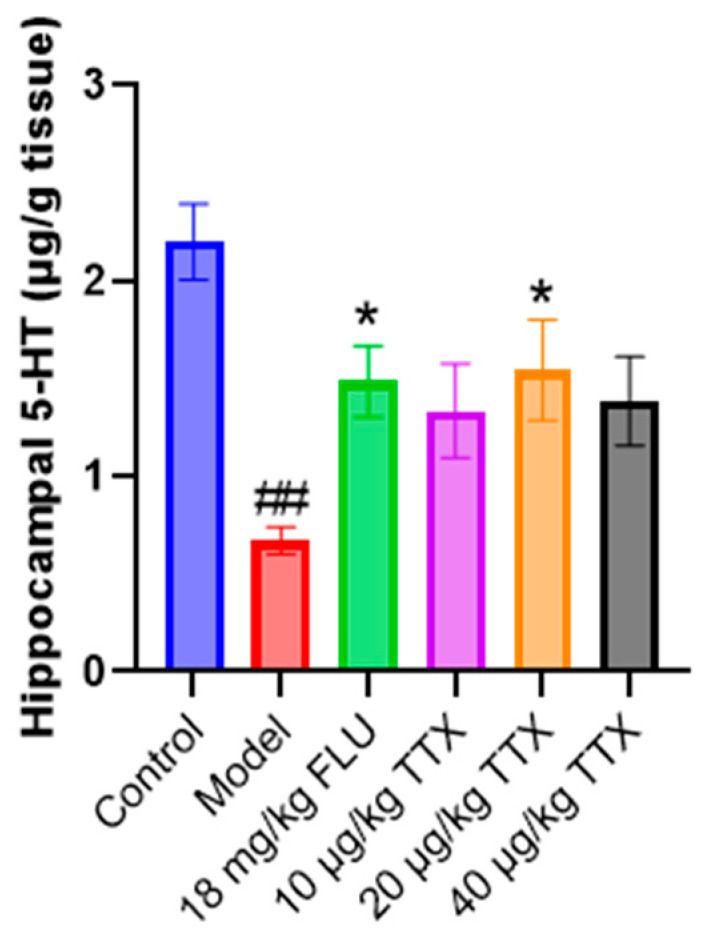
Hippocampal 5-HT content. ## *p* < 0.01 compared with the control group, * *p* < 0.05 compared with the model group.

The bar graph illustrates the 5-HT levels across different experimental groups (Figure 3). In the control group, the hippocampal 5-HT level was approximately 2.2 μg/g tissue. The model group exhibited a significant reduction in hippocampal 5-HT level, around 0.8 μg/g, suggesting a substantial depletion of 5-HT in the hippocampus under the depressive model condition. For the groups with interventions, the 18 mg/kg FLU group showed a notable increase in hippocampal 5-HT level (about 1.5 μg/g), demonstrating a significant difference compared to the model group. The groups treated with different doses of TTX oral film also presented elevated 5-HT levels, with the 20 μg/kg group showing significant differences from the model group. These results indicate that both FLU and TTX interventions can effectively increase the hippocampal 5-HT levels in the depressive model, which may contribute to their antidepressant-like effects.

### 2.4. TTX Oral Film Has No Influence on Organ Indexes

**Figure 4:**
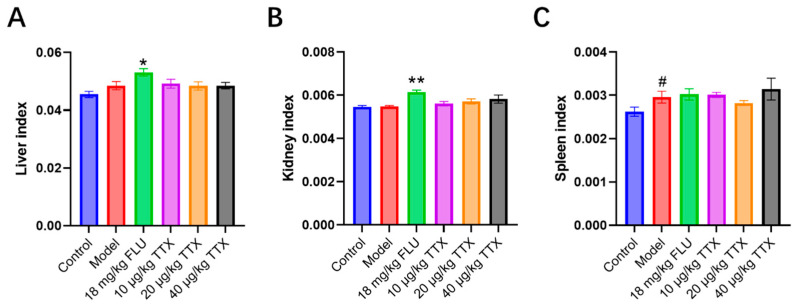
Organ indexes include: (**A**) liver index, (**B**) kidney index, and (**C**) spleen index. # *p* < 0.05 compared with the control group; * *p* < 0.05, ** *p* < 0.01 compared with the model group.

The organ indexes (liver, kidney, and spleen) of mice across different experimental groups are shown in Figure 4. At baseline, the CUMS modeling did not alter liver/kidney indexes but significantly increased the spleen index. Long-term FLU (18 mg/kg) administration led to marked elevations in liver and kidney indexes vs. model, implying potential hepatotoxicity and nephrotoxicity. In contrast, TTX (10–40 μg/kg) showed no significant effects on these organ indexes compared to model group. Thus, TTX oral films may avoid additional organ damage (beyond model-related spleen changes) with prolonged dosing.

### 2.5. Acute Toxicity of the TTX Oral Film

As shown in Table 1, mortality data were analyzed, and the LD50 was calculated by Bliss probit regression to be 919 μg/kg (95% CI: 875–965 μg/kg). During the experiment, it was observed that toxic signs emerged rapidly following dosing, characterized by progressive lethargy, marked reduction in spontaneous activity, visible peripheral vasoconstriction, tachypnea, and convulsive struggling prior to death.

## 3. Discussion

The present study demonstrated that the TTX oral film formulation produced significant antidepressant-like effects in the CUMS mouse model, effectively reversing depressive-like, anxiety-like, and cognitive impairment behaviors. Notably, this therapeutic benefit was accompanied by a restoration of hippocampal 5-HT level and a markedly improved safety profile relative to unformulated TTX.

A pivotal finding of this investigation was the significant reduction in acute toxicity achieved through the oral film formulation. The LD_50_ of the TTX oral film was determined to be 919 μg/kg, which was approximately four-fold as that reported for pure TTX (232 μg/kg) [11]. This dramatic increase underscored the critical role of pharmaceutical formulation in mitigating the inherent toxicity of potent neuroactive compounds. The observed therapeutic effects at doses as low as 20 μg/kg, merely 1/46 of the LD_50_, suggested a wide therapeutic index, a paramount consideration for clinical translation, particularly for chronic psychiatric conditions requiring long-term pharmacotherapy.

The antidepressant-like effects of TTX suggest that VGSC blockade may represent a novel mechanism for modulating the pathophysiology of depression. Although the precise molecular pathways have yet to be fully elucidated, the restoration of hippocampal 5-HT levels following TTX treatment indicated a connection between sodium channel inhibition and serotonergic neurotransmission. This finding aligns with the established understanding that depression involves dysregulation of monoaminergic systems, particularly 5-HT, and suggests that TTX may exert its antidepressant effects, at least in part, through modulation of serotonergic signaling. The hippocampal 5-HT restoration observed in our study might reflect the effect of TTX on 5-HT synthesis, release, and reuptake dynamics, warranting further mechanistic investigation into how VGSC blockade specifically influences serotonergic neuron function and synaptic plasticity in depression models. This finding is particularly intriguing in light of evidence showing that established antidepressants, such as FLU, also exhibit sodium channel blocking properties [12]. Our results support the hypothesis that reducing neuronal hyperexcitability through VGSC inhibition may represent a common, yet underrecognized, mechanism contributing to antidepressant effects. The reversal of lamotrigine’s effects by a sodium channel opener further underscores the potential role of this target in mood regulation [13].

Notably, Nav1.9, encoded by SCN11A, was found to be significantly elevated in patients with major depressive disorder compared to healthy controls, and its expression normalized after antidepressant treatment [14]. Importantly, in rat hippocampal cultures, TTX (30 nM) nearly completely suppressed glutamatergic excitatory postsynaptic currents (EPSCs) while having minimal effects on GABAergic inhibitory postsynaptic currents (IPSCs), whereas a higher concentration (1.0 μM) inhibited both EPSCs and IPSCs [15]. Given that the balance between excitatory glutamatergic and inhibitory GABAergic neurotransmission is strongly implicated in depression [16], the modulation of VGSC activity may explain the antidepressant effect of the TTX oral film in this study. This is also consistent with our finding that the 20 μg/kg dose showed the greatest efficacy.

In ventral nerve cord of the *Drosophila*, decreases in evoked 5-HT content were rescued by inhibiting action potential propagation with TTX [17]. Additionally, VGSC blockade has been reported to inhibit 5-HT release in enterochromaffin cells [18]. In mouse cerebrocortical synaptosomes, the uptake of 5-HT was decreased by inhibition of VGSC [19]. Since TTX may restore 5-HT synthesis while inhibiting its release and uptake, these mechanisms could collectively explain the regulatory effect of TTX on hippocampal 5-HT content observed in the CUMS mouse model in this study. The interplay among these opposing actions—enhanced synthesis versus inhibited release and reuptake—may ultimately result in net 5-HT level restoration in the hippocampus, though the relative contribution of each mechanism and their regional specificity require further elucidation.

From a toxicological perspective, the long-term safety profile of the TTX oral film is highly promising. Organ index is a standard toxicological parameter used to assess potential organ-specific toxicity and physiological stress in preclinical studies. Chronic administration of TTX oral film did not alter liver or kidney indexes, contrasting sharply with the significant organ weight increases observed in the FLU-treated group, which hinted at potential drug-induced organ stress. The absence of changes in liver and kidney organ indices in the TTX-treated groups suggests that chronic VGSC blockade at therapeutic doses does not induce overt hepatotoxicity or nephrotoxicity. This lack of chronic toxicity, coupled with the reduced acute toxicity, positioned the TTX oral film as a compelling candidate for further development. However, further investigation into the chronic effects of TTX is warranted. To date, no studies have comprehensively examined the long-term effects of TTX exposure. Chronic toxicity has been evaluated for another guanidine VGSC blocker, neosaxitoxin (NeoSTX), in which repeated subcutaneous administration to rats at a dose of 6 µg/kg once daily for 12 weeks resulted in reduced body weight and food intake, as well as elevated blood levels of total and direct bilirubin, gamma-glutamyl transferase, and glutamic oxaloacetic transaminase. Notably, these effects were reversible upon cessation of treatment [20]. In another study, rats administered 3 or 9 µg/L saxitoxin equivalents in drinking water exhibited altered antioxidant defenses and biochemical signs of oxidative stress in the brain and liver [21].

While this study provided robust evidence for the efficacy and safety of the TTX oral film, several limitations warrant acknowledgment. The exact subtype(s) of VGSCs mediating the antidepressant effects of TTX in the CUMS model remained unidentified. Furthermore, the specific neural circuits and downstream molecular effectors through which TTX modulated mood and hippocampal 5-HT levels were yet to be defined. Future research employing subtype-selective sodium channel modulators and detailed electrophysiological and morphological studies will be essential to unravel the precise mechanism of action.

Taken together, the TTX oral film was successfully developed, and its antidepressant effect was demonstrated in the CUMS mouse model. Behavioral tests confirmed that TTX administration alleviated core depressive and anxiety-like phenotypes, and restored cognitive performance, comparable to the conventional antidepressant FLU. Importantly, the treatment reversed the deficit in hippocampal 5-HT levels, supporting a potential role of VGSC modulation in serotonergic function.

Although these results highlight the therapeutic potential of TTX oral film as a novel antidepressant agent, its precise mechanism of action requires further investigation. Our findings support the feasibility of repurposing TTX through pharmaceutical formulation for central nervous system disorders, offering a strategically improved efficacy-safety balance for future clinical applications.

## 4. Materials and Methods

### 4.1. TTX Oral Film Preparation

TTX (purity > 99%) was provided by Third Institute of Oceanography, Ministry of Natural Resource, Xiamen, China. 0.5 g of hydroxypropyl methylcellulose (HPMC, E5 grade, Anhui Sunhere Pharmaceutical Excipients, Huainan, China) was dispersed in 2.5 mL distilled water and allowed to swell completely. Next, 0.2 g maltodextrin (Henan Qianzhi Commerce & Trade, Zhengzhou, China) and 0.2 g hydroxypropyl cellulose (HPC, LF grade, Ashland Inc., Covington, KY, USA) were added to the swollen HPMC solution with continuous stirring until complete dissolution. Subsequently, 0.2 mL of glycerin was added to the mixture. A suspension containing 0.3 g pregelatinized starch (Anhui Sunhere Pharmaceutical Excipients) and 0.36 g low-substituted hydroxypropyl cellulose (L-HPC, Anhui Sunhere Pharmaceutical Excipients) in 3 mL distilled water was prepared and incorporated into the main mixture. Then, 2.5 mL aqueous solution containing the predetermined amount of TTX was added to the polymer blend, and was stirred continuously for 30 min to ensure homogeneous drug distribution. The resulting slurry was spread uniformly on a 10 × 20 cm^2^ glass plate and subject it to drying in a constant temperature convection oven at 60 °C for 30 min. Once cooled, the dried film was carefully peeled from the glass substrate. Finally, the TTX oral film was obtained.

### 4.2. Establishment of the CUMS with Anxiety-like Depression

A total of 48 male C57BL/6J mice (6–8 weeks old, 18–22 g) were housed under specific pathogen-free conditions with controlled temperature (23 ± 2 °C), humidity (45–55%), and a 12 h light/dark cycle, with *ad libitum* access to food and water. Following a 2-week acclimatization period, all animals underwent a SPT for baseline assessment prior to group assignment. The mice were then accordingly allocated into 6 experimental groups (*n* = 8): normal control group, model group (CUMS), positive control group (FLU, 18 mg/kg), TTX 10 μg/kg group, TTX 20 μg/kg group, and TTX 40 μg/kg group.

The normal and model groups received saline via oral gavage, while the positive group received FLU (18 mg/kg) via the same route. TTX oral films were orally administered daily between 8:00–9:00 AM. The CUMS protocol consisted of multiple stressors including food/water deprivation, 45° cage tilt, physical restraint, light/dark cycle reversal, wet bedding, forced swimming in 4 °C water, tail pinching, continuous illumination, and noise exposure [22].

**Figure 5:**
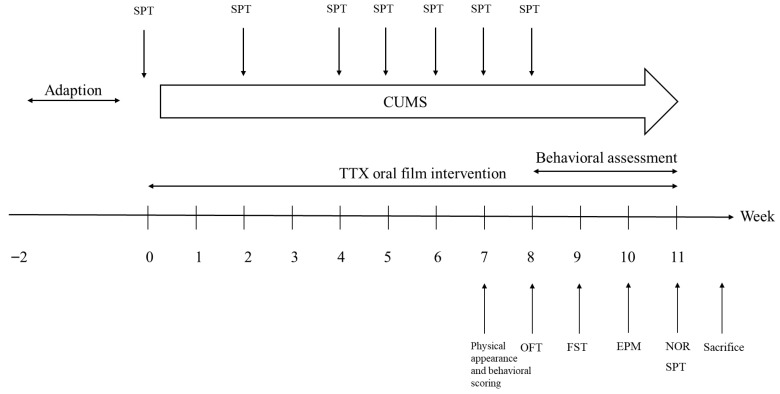
Chronological workflow of CUMS establishment, TTX oral film intervention and behavioral assessment. Values are in weeks.

Behavioral assessments were conducted at specific time points. SPT was performed once every one or two weeks to monitor the progression of anhedonia. Physical appearance and behavioral scoring were conducted on the end of the 7th Week. As illustrated in Figure 5, comprehensive behavioral evaluations, including the OFT, FST, EPM, NOR, and SPT were performed between the 8th and 11th Weeks.

### 4.3. Physical Appearance and Behavior Scoring

Depression-like behaviors in mice were quantitatively assessed using a standardized phenotypic scoring system (maximum score = 10 points) based on two key clinical parameters:

I. Fur condition (6 points total)

Pigmentation (5 points): Five body regions (limbs, head, neck, back, and tail) were evaluated, with 1 point awarded for each region maintaining normal black/shiny appearance and 0 points for yellowish discoloration.

Alopecia (1 point): 1 point for normal fur density, 0 points for visible hair loss.

II. Behavioral responsiveness (4 points total)

Capture resistance (1 point): 1 point for normal evasive behavior during handling, 0 points for passive acceptance.

Stress response to gavage (3 points): 3 points for intense struggle or screams; 2 points for mild struggle with screams; 1 point for slight struggle that stops immediately; 0 points for no resistance.

This comprehensive scoring system enabled objective quantification of depression-related phenotypic alterations, where higher scores correlated with better physical and behavioral status. All evaluations were conducted by experimenters blinded to treatment groups to eliminate assessment bias.

### 4.4. Behavioral Assessments

The SPT consisted of both training and testing phases. During the initial two-day training period, mice were habituated to sucrose by providing two bottles containing 1% sucrose solution on Day 1. On Day 2, one sucrose bottle was replaced with plain water, with bottle positions reversed after 6 h to control for side preference. For the subsequent testing phase, following an 18 h fasting period, animals were subjected to a 2 h preference test with simultaneous access to one bottle of 1% sucrose solution and one bottle of plain drinking water. Bottle positions were switched after 1 h to minimize positional bias. Liquid consumption was determined by pre- and post-test weighing of both bottles, enabling calculation of the sucrose preference ratio (sucrose solution intake divided by total liquid intake).

The OFT was conducted using an automated behavioral tracking system (EthoVision XT 8.0, Noldus Information Technology, Wageningen, Netherlands). Prior to testing, all animals received their scheduled administration and were acclimated to the testing environment for 60 min to minimize stress-induced variability. For the actual assessment, individual mouse was gently placed into the enclosed open field arena in a consistent orientation. Following a 30 s habituation period to allow for initial environmental exploration, each mouse’s locomotor activity was video-recorded for a 5 min test session. The primary behavioral parameter analyzed was the time spent exploring the central zone of the arena, a validated measure of anxiety-like behavior in rodents. Between trials, the apparatus was thoroughly cleaned with 70% ethanol to eliminate olfactory cues that might influence subsequent animals’ behavior.

The FST was performed in a cylindrical polypropylene tank (height 40 cm, diameter 12 cm) containing 10 cm of water maintained at 25 ± 1 °C. Each mouse was gently lowered into the apparatus and recorded for 6 min. The first 2 min served as habituation; the final 4 min were analyzed for immobility, defined as passive floating with only the minimal limb movements required to keep the head above water (e.g., occasional single-limb paddling or subtle postural adjustments without active struggling). Immobility duration was automatically quantified with EthoVision XT 8.0 (Noldus Information Technology). After each trial, the water was replaced and the tank thoroughly cleaned to ensure consistent conditions across subjects.

For the EPM test, an infrared camera was positioned above the apparatus and activated prior to testing. Each mouse was placed in a closed arm with its head oriented toward the end wall. After a 30 s acclimation period, behavior was recorded for 5 min and subsequently analyzed with EthoVision XT 8.0 (Noldus Information Technology). The primary anxiety-related metric was the percentage of time spent exploring the open arms.

The NOR test was conducted in an open-field arena through three consecutive phases. Mice first underwent a 5 min habituation period to explore the empty arena. In the familiarization phase, two identical objects (A1, A2) were placed 5 cm from opposite walls for 5 min of exploration. For the test phase, one familiar object was replaced with a novel object (B1) while maintaining the same spatial configuration, with exploration recorded for another 5 min. The recognition index was calculated as: Time exploring B1/Time exploring (B1 + A1) × 100%. EthoVision XT 8.0 (Noldus Information Technology) was used for behavioral recording and analysis.

### 4.5. Calculation of Organ Indexes and Separation of Hippocampal Samples

Following 11 weeks of drug administration and the completion of behavioral testing, the animals were euthanized for tissue collection. This was performed one hour after the final drug dose by an overdose of inhaled isoflurane, followed by cervical dislocation. The liver, spleen, and kidney were carefully dissected and weighed, and the organ index was calculated as the ratio of organ weight to body weight. Hippocampal tissues were rapidly dissected on an ice-cold platform, and then flash-frozen in liquid nitrogen and stored at −80 °C for subsequent analyses. For biochemical assays, hippocampal tissues were homogenized in ice-cold PBS at a ratio of 1:9 (*w*/*v*) using ultrasonic disruption, followed by centrifugation at 12,000× *g* for 15 min at 4 °C. The resulting supernatant was used to determine 5-HT levels following the manufacturer’s instructions (Nanjing Jiancheng Bioengineering Institute, Nanjing, China).

### 4.6. Acute Toxicity

A total of 60 Kunming mice (30 males and 30 females) were acclimatized for 3 days prior to experimentation. Based on preliminary dose-range findings, 5 geometric dose levels were selected between 500–1000 μg TTX/kg body weight: 500, 588, 692, 814 and 958 μg/kg, along with a vehicle control. All doses were computed from the TTX content in the oral film formulations. The animals were randomly assigned to the above groups. Each mouse received a single oral dose and was monitored continuously for 14 days.

### 4.7. Statistical Analysis

For the behavioral studies, data were presented as mean ± SEM. Histograms were created using GraphPad Prism 9 software (GraphPad Inc., San Diego, CA, USA), and statistical analysis were performed with one-way ANOVA followed by Tukey’s multiple comparison test using the same software. Significant differences were considered when *p* value was less than 0.05.

For the acute toxicity study, statistical analysis of the experimental data was performed using SPSS 17 software (IBM Corp., Armonk, NY, USA), and the Bliss method was used to calculate the LD50.

## Figures and Tables

**Table 1:** Results of the acute toxicity test of TTX oral film.

Groups	Deaths/Animals		
	♀	♂	Total
Control	0/5	0/5	0/10
500 μg/kg TTX oral film	0/5	1/5	1/10
588 μg/kg TTX oral film	0/5	0/5	0/10
692 μg/kg TTX oral film	1/5	1/5	2/10
814 μg/kg TTX oral film	1/5	0/5	1/10
958 μg/kg TTX oral film	4/5	5/5	9/10

## Data Availability

Data will be made available on request.

## Abbreviations

The following abbreviations are used in this manuscript:

TTX	Tetrodotoxin
VGSCs	Voltage-gated sodium channels
CUMS	Chronic unpredictable mild stress model
FLU	Fluoxetine
SPT	Sucrose preference test
OFT	Open field test
FST	Forced swimming test
NOR	Novel object recognition
EPM	Elevated plus maze
5-HT	Serotonin
NeoSTX	Neosaxitoxin
